# Postallograft aleukemic mast cell leukemia, with macrophagic activation syndrome

**DOI:** 10.1002/jha2.431

**Published:** 2022-04-26

**Authors:** Salwa Hamdash, Corentin Deckers, Virginie Chapelle, Martin Vanderdonck, Pascale Saussoy, Madeleine Rousseaux

**Affiliations:** ^1^ Haematology Department of Laboratory Medicine Cliniques Universitaires Saint‐Luc Brussels Belgium; ^2^ Genetics Department of Laboratory Medicine Cliniques Universitaires Saint‐Luc Brussels Belgium

1

A 60‐year‐old male suffering from hypertension and a treated myelodysplastic syndrome (MDS‐EB2) by an allograft 3 years before the current presentation was presented with a deteriorating general condition and asthenia. A blood work‐up was done where hypereosinophilia (24 × 10^3^/μl) was incidentally found, hardly attributable to a postallogeneic peripheral blood stem cell transplantation [2].The patient was then referred to our hematology department for further investigation. Upon admission, a myelogram revealed 7% of mastocytes, often in aggregates, some showing atypical forms (hypogranulation, spindle‐shape, and hypersegmentation of nucleus) (Figure 1A,B), and 51% of eosinophilic lineage. Flow cytometry (FC) showed 7% of mast cells with CD2 and CD25 aberrant expression. No genetic abnormalities including typical c‐KIT mutation were shown by NGS (whole exome) and PCR (only mutation c.2447A>T (p.D816V)). Fluorescence in situ hybridization (FISH) technique showed no translocation involving FIP1L1, PDGFRA, or PDGFRB. Molecular chimerism was complete with a sensitivity threshold of 5%. With major criteria, the diagnosis of systemic mastocytosis with eosinophilia was retained. This diagnosis was supported by hepatosplenomegaly and signs of symmetrical medullary recruitment in the long bones. Given the patient's initial diagnosis, serum tryptase (higher than 20 ng/ml) could not be used as a valid minor criterion [1, 3].

**FIGURE 1:**
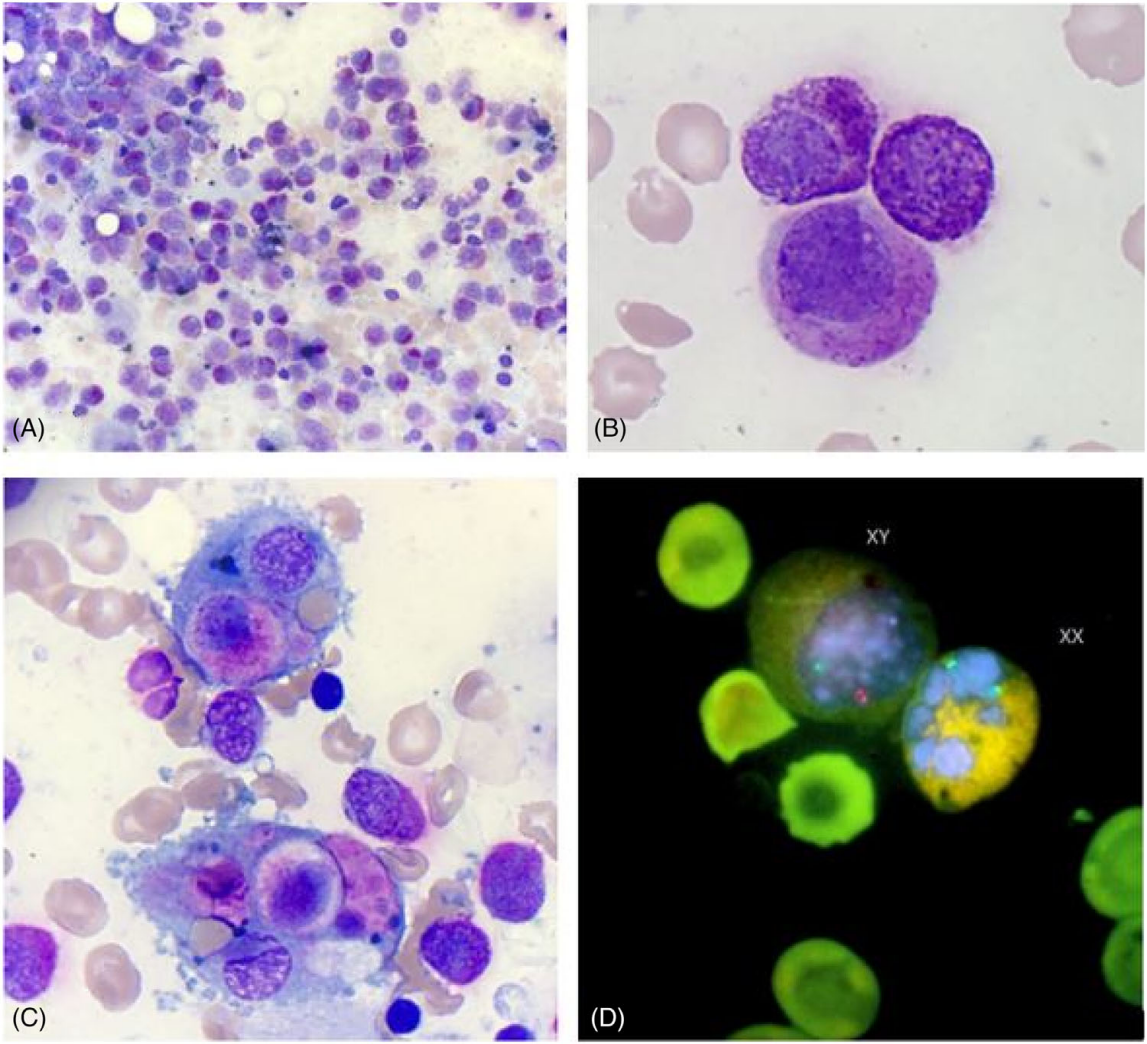
(A) Significant infiltration by atypical mast cells in second marrow aspiration, May‐Grünwald‐Giemsa stain, original magnification 10×. (B)  Hypogranulated mast cell with eccentric nucleus, original magnification 50×. (C) Two images of multilineage hemophagocytosis, original magnification 100×. (D) An atypical XY (recipient) mast cell and an XX (donor) neutrophil, original magnification 100×

Two weeks later, the patient presented rapid clinical deterioration with severe hypotension, a subfebrile state at 37.7°C, and a poorly tolerated rapid ventricular fibrillation, motivating his transfer to the intensive care unit. To our knowledge, no precedenting history (e.g. COVID and viral infection) could predispose this patient for precipating the macrophagic activation syndrome. Repeated blood tests showed worsening thrombocytopenia (6 × 10^3^/μl), hyperferritinemia (9685 μg/L), elevated fibrinogen (452 mg/dl), hypertriglyceridemia (360 mg/dl), and altered liver function tests. Thus, we performed a second marrow aspiration to clarify the diagnosis. Cytology reported 57% of atypical mature mastocytes (hypogranulated or degranulated forms, eccentric nucleus), only 4% of eosinophils and a secondary hemophagocytic lymphohistiocytosis (Figure 1C). FC showed 39% of mastocytes with CD25 expression alone, without CD2. Circulating mastocytes represent 2%, confirming an aleukemic form of MCL (blood mastocytes < 10%). The patient was treated by imatinib (Glivec, Novartis) 200 mg per day. He was then intubated, and received packs of blood and platelets as supportive treatment. Unfortunately, he died 2 weeks after the diagnosis of mast cell leukemia. Molecular biology identified mixed chimerism on the second BM, with 24% of the recipient.

To analyze the possible origin of the relapse (donor versus recipient), a third post‐mortem myelogram was done, where we compared chimerism by molecular biology and by cytogenetic with FISH technique. The third BM showed also mixed chimerism by molecular biology with 17% of the recipient.

Cells of the second BM analyzed by FISH were obtained by culture in a medium not stimulated by growth factors [4, 5].

This medium is suitable for mast cell growth. Cytogenetics targeted the cells by FISH with probes directed against gonosomes X and Y. The male patient (XY) received an allograft stem cell transplant from a female donor (XX) which made it possible to make an easy distinction between the cells of the donor and those of the recipient.

The probes allowed the detection of 8.5% of cells exhibiting a Y chromosome, which gives a result of mixed chimerism with predominance of the donor (91% of the cells analyzed).

Next, we combined mast cell morphology with cytogenetic to prove that mast cells correspond to either XX donor or XY receiver. For this purpose, we used FISH technique on slides from the second BM. Classical FISH treatment allowed complete preservation of the nucleus and cytoplasm of the cells, and thus morphological recognition of atypical mast cells can easily be done. The abnormal mast cells clearly showed a male origin (XY). While the megakaryocyte, erythrocyte, and granulocyte lineages showed a female origin (XX) (Figure 1D). Leukemic cells are therefore of recipient origin, which coincides with the concomitant loss of chimerism observed by molecular biology.

Mast cell leukemia is a rare disease with extremely poor diagnosis, with survival rate of <1 year in most patients [1]. Despite his treatment, our patient developed multiple organ failure, leading to prioritizing his comfort.

In conclusion, this case report highlights the importance of a close up cytological monitoring in postallograft myeloid neoplasms with hypereosinophilia.

## CONFLICT OF INTEREST

The authors declare no conflict of interest.

## FUNDING INFORMATION

None.

## AUTHOR CONTRIBUTIONS

Salwa Hamdash, Corentin Deckers, and Virginie Chapelle collected, analyzed the data, and performed the diagnosis with Madeleine Rousseaux. Salwa Hamdash, Corentin Deckers, Virginie Chapelle, and Martin Vanderdonck wrote the manuscript and cover letter. Madeleine Rousseaux and Pascale Saussoy reviewed the manuscript. All authors have reviewed and approved the manuscript.
